# Plasticity of the hypocretinergic/orexinergic system after a chronic treatment with suvorexant in rats. Role of the hypocretinergic/orexinergic receptor 1 as an autoreceptor

**DOI:** 10.3389/fnmol.2022.1013182

**Published:** 2022-10-05

**Authors:** Carlos Carrera-Cañas, Isabel de Andrés, Marta Callejo, Miguel Garzón

**Affiliations:** Departamento de Anatomía, Histología y Neurociencia, Facultad de Medicina, Universidad Autónoma de Madrid, Madrid, Spain

**Keywords:** hypocretin, orexin, narcolepsy, DORAs, suvorexant, hypothalamus

## Abstract

The hypothalamic hypocretinergic/orexinergic (Hcrt/Ox) system is involved in many physiological and pathophysiological processes. Malfunction of Hcrt/Ox transmission results in narcolepsy, a sleep disease caused in humans by progressive neurodegeneration of hypothalamic neurons containing Hcrt/Ox. To explore the Hcrt/Ox system plasticity we systemically administered suvorexant (a dual Hcrt/Ox receptor antagonist) in rats to chronically block Hcrt/Ox transmission without damaging Hcrt/Ox cells. Three groups of eight rats (four males and four females) received daily i.p. injections of suvorexant (10 or 30 mg/kg) or vehicle (DMSO) over a period of 7 days in which the body weight was monitored. After the treatments cerebrospinal fluid (CSF) Hcrt1/OxA concentration was measured by ELISA, and hypothalamic Hcrt/OxR1 and Hcrt/OxR2 levels by western blot. The systemic blockade of the Hcrt/Ox transmission with the suvorexant high dose produced a significant increase in body weight at the end of the treatment, and a significant decrease in CSF Hcrt1/OxA levels, both features typical in human narcolepsy type 1. Besides, a significant overexpression of hypothalamic Hcrt/OxR1 occurred. For the Hcrt/OxR2 two very close bands were detected, but they did not show significant changes with the treatment. Thus, the plastic changes observed in the Hcrt/Ox system after the chronic blockade of its transmission were a decrease in CSF Hcrt1/OXA levels and an overexpression of hypothalamic Hcrt/OxR1. These findings support an autoregulatory role of Hcrt/OxR1 within the hypothalamus, which would induce the synthesis/release of Hcrt/Ox, but also decrease its own availability at the plasma membrane after binding Hcrt1/OxA to preserve Hcrt/Ox system homeostasis.

## Introduction

The hypocretinergic/orexinergic (Hcrt/Ox) system is a neuromodulatory network involved in several physiological processes including feeding and sleep/wakefulness behaviors, and endocrine and autonomic functions (Kukkonen et al., 2002). It consists of two excitatory neuropeptides known as Hcrt1 and Hcrt2, or OxA and OxB (De Lecea et al., 1998; Sakurai et al., 1998), which are produced specifically by a small group of hypothalamic neurons mainly concentrated in the perifornical area within the posterior and lateral hypothalamus (De Lecea et al., 1998; Sakurai et al., 1998; Nuñez et al., 2006; García-García et al., 2013). These neurons project to multiple target areas in the central nervous system (Peyron et al., 1998) where Hcrt/Ox peptides bind two G protein-coupled receptors: Hcrt/OxR1, with a much higher affinity for Hcrt1/OxA, and Hcrt/OxR2, which binds to both Hcrt/Ox peptides indistinctly (Sakurai et al., 1998). The deficit of Hcrt/Ox neurotransmission is associated with human narcolepsy (Nishino et al., 2000), and with other pathophysiological processes (Emamzadeh and Surguchov, 2018; Wang et al., 2018).

Narcolepsy is a rare neurological disorder associated with a significant dysregulation of the sleep-wakefulness cycle, in which the control of rapid eye movement (REM) sleep is lost. Clinical characteristics include excessive daytime sleepiness and the intrusion of REM sleep traits into wakefulness, such as transient loss of muscle tone (cataplexy), hypnagogic/hypnopompic hallucinations or sleep paralysis (Bassetti et al., 2019). Beyond the symptoms associated with sleep, narcoleptic patients also present many autonomic and metabolic/endocrine disturbances, including an increased body-mass index (Schuld et al., 2000; Plazzi et al., 2011). Narcolepsy is currently classified into type 1 (NT1) and type 2 (NT2) (American Academy of Sleep Medicine [AASM], 2014), whose main differential clinical characteristic is that cataplexy (lack of muscle tone) occurs in NT1, but not in NT2. Narcoleptic type I patients present a global loss of Hcrt/Ox neurons that is reflected in decreased levels of Hcrt1/OxA in cerebrospinal fluid (CSF), and also by an absence of signal for Hcrt/Ox immunostaining in the hypothalamus (Nishino et al., 2000; Peyron et al., 2000; Mignot et al., 2002).

There are several animal models of narcolepsy -including canines and rodents- with different pathophysiology and characteristics (Chen et al., 2009). Genetic models mimic to differing extents some of the clinical features of human narcolepsy, although narcolepsy in humans is not directly linked to genetic alterations in the Hcrt/Ox system. Genetic models do not enable the examination of the Hcrt/Ox system adaptability during the development and progression of the disease. By contrast, chronic pharmacological models can express progressive changes in a dose-dependent manner (Winrow et al., 2011), and consequently they are suitable for the exploration of the homeostatic performance of the Hcrt/Ox system to compensate its alterations.

Many studies have focused on the pathophysiological mechanisms mediated by the Hcrt/Ox system (Peyron et al., 2000; Thannickal et al., 2000; España et al., 2001; Mignot et al., 2002; Mochizuki et al., 2004; Kantor et al., 2009; Diniz-Behn et al., 2010; Hansen et al., 2017), but its self-regulatory physiological functioning is still poorly understood. The complete pharmacological blockade of the two Hcrt/Ox receptors in mice lead to an experimental model with narcolepsy-like features (Mahoney et al., 2020; Kaushik et al., 2021), but with undamaged Hcrt/Ox cells. Then, this latter model could be useful to explore plasticity mechanisms of the Hcrt/Ox system under physiopathological conditions and get insight also into adaptive changes present at initial stages of narcolepsy before total loss of Hcrt/Ox neurons. In the present study we have accomplished a chronic systemic administration of two doses of suvorexant (a dual Hcrt/Ox receptor antagonist) in rats to block both Hcrt/Ox receptors, i.e., Hcrt/OxR1 and Hcrt/OxR2. We hypothesized that as long as the Hcrt/Ox neurons continue to maintain their functionality, homeostatic mechanisms would be expressed which would attempt to compensate for the alterations produced by the exogenous blockade of the Hcrt/Ox receptors. To test this hypothesis, and get insight into self-regulating Hcrt/Ox mechanisms, we measured the CSF levels of Hcrt1/OxA and the expression of Hcrt/OxR1 and Hcrt/OxR2 in the posterior hypothalamic tissue after suvorexant/vehicle administration. Changes in the body weight of the animals were also analyzed to evaluate the effectiveness of the suvorexant treatment (Schuld et al., 2000).

## Materials and methods

### Subjects and surgery

Twenty-four adult Sprague Dawley rats (12 males and 12 females) aged between 4 and 5 months were used in this study. The animals were housed in pairs in a temperature and humidity-controlled room with water and food *ad libitum* and on a reverse 12:12-h light/dark cycle (lights on at 12:00 p.m., and off at 12:00 a.m.). All the experiments were carried out in accordance with the European Community Council Directive (2010/63/UE) and approved by the Institutional Animal Care and Use Committee of the Universidad Autónoma de Madrid (Spain) and the competent regional government agency (PROEX 131.8/20).

The animals were divided into three experimental groups each with eight rats (four males and four females). Suvorexant (MK-4305, Biorbyt) was dissolved through vortexing in 100% dimethyl sulfoxide (DMSO; D8418, Sigma-Aldrich, Darmstadt, Germany) (Simmons et al., 2017) to get doses of 10 or 30 mg/kg for each animal, depending on the experimental group to which it belongs. All the treatments were carried out in a period of approximately 1 month, with a random distribution of the animals of the different experimental groups. They consisted of 2 days of vehicle administration in the three groups, followed by either 7 days of suvorexant (10 or 30 mg/kg) or vehicle administration (control group) (Winrow et al., 2011). All the compounds were administered i.p. daily at a fixed volume of 150 μl 2 h before the lights were turned on. Body weight was monitored starting from the day prior to the treatments, before injecting the vehicle in the three groups of rats (day 0), and throughout the treatments with DMSO and the two doses of suvorexant, on days 4, 7, and 10.

### Cerebrospinal fluid and posterior hypothalamic tissue sample collections

The day after the last injection (day 10), animals were anesthetized i.p. with 1.4 g/Kg urethane (U2500, Sigma-Aldrich, Darmstadt, Germany) at the end of the dark phase. CSF was collected by puncture of the cisterna magna and frozen immediately, and the animals were decapitated for brain extraction. The posterior hypothalamic area was carefully dissected out over ice and immediately homogenized in RIPA buffer. All the samples were stored at −80°C until subsequent analyses.

### Cerebrospinal fluid determination of Hcrt1/OxA levels

The CSF Hcrt1/OxA concentrations were measured by a competitive enzyme-linked immunosorbent assay (ELISA) with a commercially available kit (EK-003-30, Phoenix Pharmaceuticals, Karlsruhe, Germany). Working with duplicates, 50 μl of standard peptide solutions or CSF were directly dispensed into secondary antibody precoated wells and incubated together with 25 μl of biotinylated Hcrt1/OxA-peptide and 25 μl of primary antibody at room temperature on a shaker for 2 h. After washing, 100 μl of Streptavidin-horseradish peroxidase (SA-HRP) were added to each well for 1 h incubation, and the plate was washed again. 3,3′,5,5′-Tetramethylbenzidine (TMB) substrate solution (100 μl) was added and incubated for 1 h. The reaction was stopped with 100 μl of 2 N hydrochloric acid, and the absorbance was read at 450 nm within 20 min. The intensity of the colorimetric reaction was directly proportional to the amount of the biotinylated Hcrt1/OxA-peptide–SA-HRP complexes and inversely proportional to the amount of Hcrt1/OxA in the standard solutions, or in the CSF. The Hcrt1/OxA concentration in the CSF samples was calculated by extrapolation of the absorbance values on the standard sigmoid curve equation (Hcrt1/OxA standard solutions range: 0.01–100 ng/ml).

### Protein expression of hypocretin/orexin receptors in the posterior hypothalamus

The hypothalamic tissue was mechanically homogenized in RIPA buffer (pH = 7.4) for western blot analysis. The protein concentration of the samples was determined using the Pierce™ BCA Protein Assay Kit (23,225, ThermoFisher Scientific, Waltham, MA, USA). A denaturing and reducing Laemmli buffer was mixed with 20 μg of sample extract, and resolved by SDS-PAGE. Proteins were electrotransferred onto a 0.45 μm pore size nitrocellulose membrane (GE10600002, Sigma-Aldrich, Darmstadt, Germany), that was blocked with 10% milk in phosphate buffered saline with 0.1% tween (0.1% PBS-T). The membrane was incubated with primary antibodies against Hcrt/OxR1 (ab68718, Abcam, Cambridge, UK 1:200), Hcrt/OxR2 (ab183072, Abcam, 1:300), and cofilin (ab54532, Abcam, 1:300) for 48 h at 4°C with gentle shaking. The specificity of the primary antibodies was proved in all the experiments by including a negative control sample (liver tissue, which does not express the Hcrt/OxRs). Biotin-conjugated secondary antibodies (1:400) were incubated with the membrane for 1 h at room temperature on a shaker, and later the membrane was incubated with ABC elite complex (PK-6100, Vector Laboratories, Burlingame, CA, USA) under the same conditions as that carried out for the peroxidase staining. For signal measurements, the enhanced chemiluminescence detection system (ECL, GERPN2106, Sigma-Aldrich, Darmstadt, Germany) and the Chemi-DOC XRS + system (BioRad, Hercules, CA, USA) were used. The quantification of the bands was performed by densitometry with the Image Lab software under conditions of non-saturated signal to ensure the linearity range. Hcrt/OxR1 and Hcrt/OxR2 signals were normalized against those of cofilin (loading control) for comparative analyses.

### Statistical analysis

Non-parametric statistics were used including the Friedman analysis of variance and the Wilcoxon matched pairs signed-ranks test for analysis of the changes in body weight after the different treatments and comparison of the expression levels of hypothalamic Hcrt/OxR1 and Hcrt/OxR2 in the control group. The Kruskal–Wallis one-way analysis of the variance and the Mann–Whitney *U* test were used to investigate the plastic changes in hypothalamic Hcrt/OxR1 and Hcrt/OxR2 expression, as well as in the CSF Hcrt1/OxA levels, which might have occurred as consequence of the different experimental treatments. The relative changes in body weight occurring within the 0–4, 4–7, and 7–10 day intervals were obtained in order to know the time course of the body weight changes through the experiments, and a parametric two-way ANOVA with replicated design (time intervals × treatment × subject) was used for comparisons between vehicle and suvorexant administration. Also, to know the effects of the different treatments in relation with the sex of the animals and the interaction between the two factors, the data in males and females in the dimethyl sulfoxide (DMSO) and the 30 mg/Kg of suvorexant groups were normalized as percentages, and two-way parametric ANOVAs with replicated design (treatment × sex × subject) were applied. *Post hoc* multiple pair analyses were performed using Fisher’s pairwise comparison test. Statistical significance was set at *p* ≤ 0.05.

## Results

### Body weight changes associated with treatments

**FIGURE 1:**
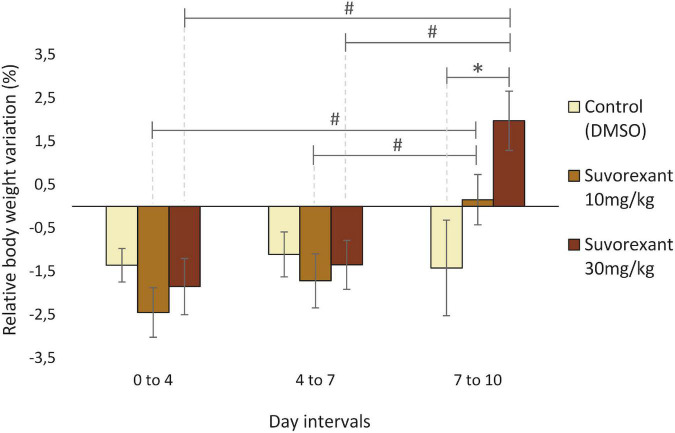
Relative body weight variation throughout the treatments. Bar graphs show average percentage values (mean ± standard error) of change in body weight relative to the first day in each time interval (0–4, 4–7, and 7–10 days) for the three treatments (DMSO, suvorexant 10 and 30 mg/kg/day). *p ≤ 0.05 (Fishers Least Significant Difference test), suvorexant 30 mg/kg vs. vehicle. ^#^*p* ≤ 0.05 (Fishers Least Significant Difference test), suvorexant 7–10 day interval vs. suvorexant 0–4 and 4–7 day intervals.

Given the close relationship between the Hcrt/Ox system and the feeding behavior and metabolism, we measured the body weight variation to evaluate the effectiveness of the chronic treatment with suvorexant in our rats. All the groups had a moderate but statistically significant decrease in body weight at the end of the treatments, as indicated by the different Friedman ANOVAs (Control group: χ^2^ = 19.05, df = 3, *p* = 0.001, median day 0 = 318.8, median day 10 = 296.55; suvorexant 10 mg/Kg group: χ^2^ = 18.6, df = 3, *p* = 0.003, median day 0 = 318.8, median day 10 = 325.2; suvorexant 30 mg/Kg group: χ^2^ = 10.95, df = 3, *p* = 0.012, median day 0 = 346.1, median day 10 = 340.9). However, the time course of the changes in body weight was different under the three different treatments. This became evident when comparing the differences in body weight relative to the values of the first day in each interval that occurred within the 0–4; 4–7; and 7–10 day intervals. A two-way replicated design ANOVA showed statistically significant differences not only between the different treatments [*F*_(2,71)_ = 3.828; *p* ≤ 0.05], but also between the different time intervals [*F*_(2,71)_ = 7.369, *p* ≤ 0.005]. Multiple pair-contrasts using the Fisher test indicated that in the control group the rate of weight loss did not present statistically significant differences between the three time intervals. Therefore, these animals exhibited a progressive weight loss that was similar throughout the entire period they received vehicle injections (Figure 1). However, this was not the case during the treatments with suvorexant. As shown in Figure 1, during the last interval (days 7–10), the animals gained weight in a statistically significant manner (*p* ≤ 0.05) when compared to the decrease in weight that was recorded during the previous intervals. Nevertheless, statistical significance for the increase in weight occurring during the last interval of treatment was only observed in the high dose group when compared with the values of the control group (Figure 1).

### Molecular changes in the hypocretinergic/orexinergic system induced by suvorexant

To address the plasticity of the Hcrt/Ox system itself under conditions of progressive loss of Hcrt/Ox transmission, as it occurs in human NT1, we analyzed the expression changes in the Hcrt1/OxA peptide, as well as in the hypothalamic Hcrt/Ox receptors after the treatments.

#### Cerebrospinal fluid Hcrt1/OxA levels

**FIGURE 2:**
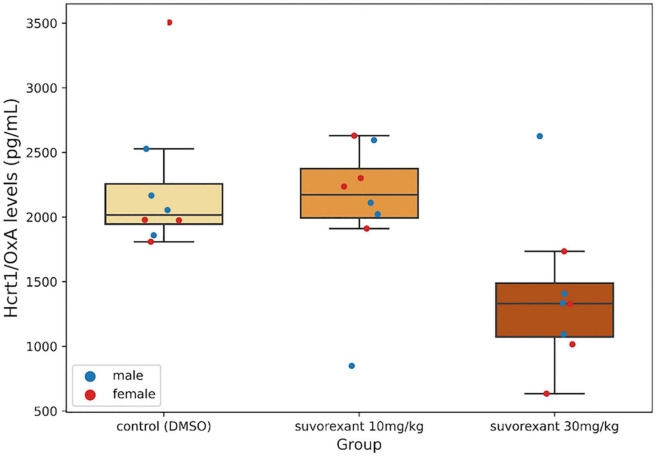
Cerebrospinal fluid (CSF) Hcrt1/OxA levels. Boxplots show the values for the CSF Hcrt1/OxA concentration in the different experimental groups. Each dot represents a single Hcrt1/OxA measure in CSF for the four males and the four females in each group. The three horizontal lines of the boxes represent the first, second (median), and third quartiles, respectively, with the whisk extending to 1.5 inter-quartile range.

The Krustal–Wallis analysis of variance showed statistically significant differences between the three treatments (*H* = 7.28, df = 2, *p* = 0.027). *Post hoc* comparisons with the Mann–Whitney *U* test indicated that the change following the low dose of suvorexant did not reach statistically significant values when compared with the control group (*Z* = −0.525, *p* = 0.599). Only the high dose of suvorexant produced a significant decrease in Hcrt1/OxA in CSF by comparison with both the control (*Z* = −2.626, *P* = 0.009) and the 10 mg/kg groups (*Z* = −1.995, *p* = 0.046) (Figure 2).

#### Protein expression levels of the hypocretin/orexin receptors in the posterior hypothalamus

**FIGURE 3:**
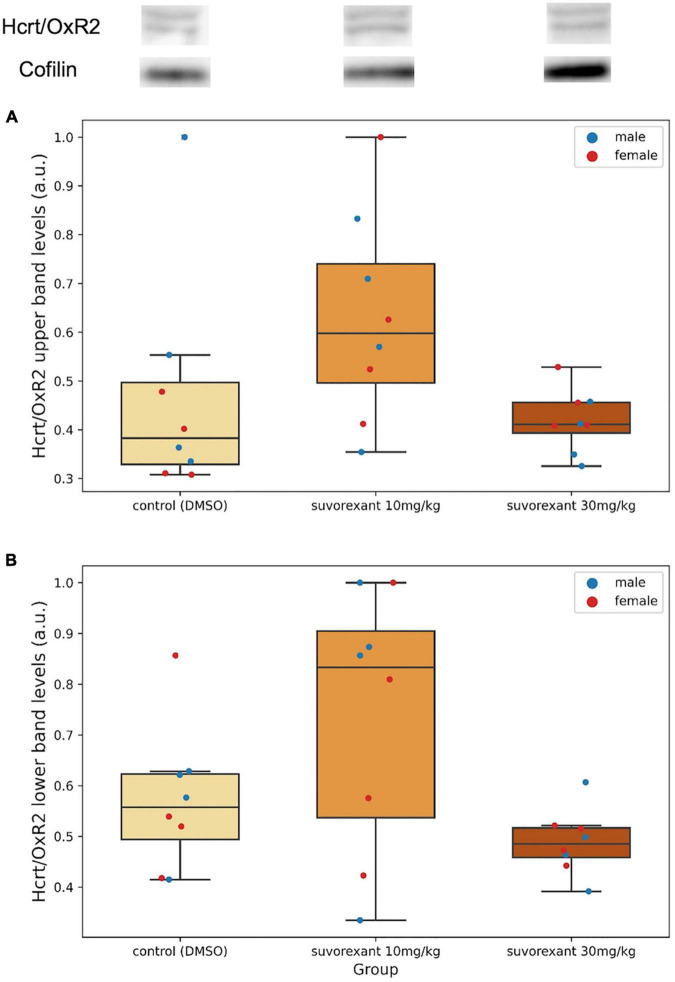
Upper part depicts examples of western blot membranes for Hcrt/OxR2 and cofilin in hypothalamic extracts from each experimental group. Note the presence of two very close bands for this receptor (see also Supplementary Figure 2). **(A,B)** Are boxplots showing the relative hypothalamic values of the upper band **(A)** and the lower band **(B)** levels of the Hcrt/OxR2 normalized to cofilin for each group. Dots represent single measures of the levels of the upper band **(A)** and the lower band **(B)** of the Hcrt/OxR2 normalized to cofilin for the males and the females in each group. The three horizontal lines of the boxes represent the first, second (median), and third quartiles, respectively, with the whisk extending to 1.5 inter-quartile range.

After western blot analysis, in relation to Hcrt/OxR2, two very close bands (see Hcrt/OxR2 in Figure 3, upper part; Supplementary Figure 2) were detected in the membrane around the area of 50 KDa, corresponding to the anticipated size of the receptor. Since these bands were absent in the negative control sample -liver homogenate- (Supplementary Figure 2), which does not express the Hcrt/Ox receptors, they must be specific for the Hcrt/OxR2.

**FIGURE 4:**
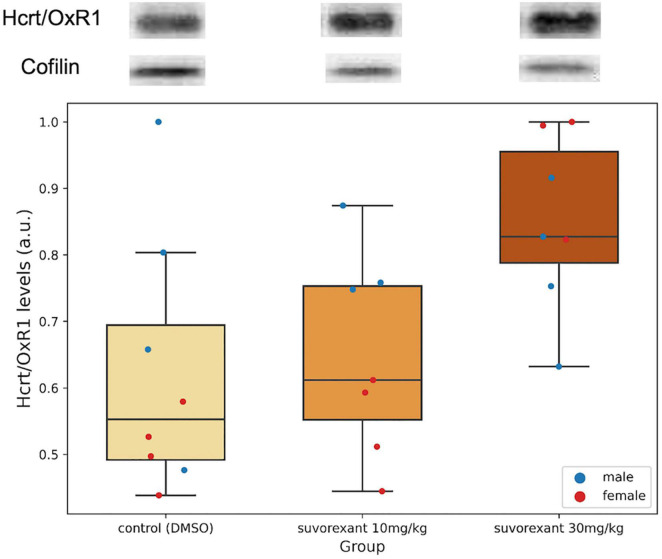
Hcrt/OxR1 hypothalamic levels. Western blots for Hcrt/OxR1 and cofilin in hypothalamic extracts from each experimental group. Boxplots show the relative hypothalamic values of Hcrt/OxR1 levels normalized to cofilin for each group. Dots represent single measures of Hcrt/OxR1 levels normalized to cofilin for the males and the females in each group. The number of subjects for the Hcrt/OxR1 analysis in the experimental groups is seven due to the lack of tissue availability in one subject. The three horizontal lines of the boxes represent the first, second (median), and third quartiles, respectively, with the whisk extending to 1.5 inter-quartile range.

A first comparison in the control group between the protein levels of Hcrt/OxR1 and Hcrt/OxR2 (added values of both bands) in the posterior hypothalamic tissue was carried out. All animals (males and females) presented a higher amount of Hcrt/OxR1 (median = 1.62, range = 1.29–3.11 a.u.) than Hcrt/OxR2 (median = 0.89, range = 0.40–1.19 a.u.). The difference between hypothalamic Hcrt/OxR1 and Hcrt/OxR2 expression was statistically significant (Wilcoxon signed-rank test: *Z* = 2.521, *p* = 0.011). Plastic changes were observed in the hypothalamic Hcrt/Ox receptors as consequence of the treatments. With regard to the Hcrt/OxR1 levels, the Krustal–Wallis analysis of variance comparing the control and the two suvorexant groups revealed significant changes (*H* = 6.378, *p* = 0.041). *Post hoc* pair contrasts with the Mann–Whitney *U* test (Figure 4 and Supplementary Figure 1) showed similar expression values of this receptor between the control and the low-dose groups (*Z* = 0.521, *p* = 0.602). Only the high dose of suvorexant produced an overexpression of the hypothalamic Hcrt/OxR1 that was statistically significant compared with the values for both the control and the low-dose groups (*Z* = 2.003, *p* = 0.045, and *Z* = 2.175, *p* = 0.030, respectively) (Figure 4). In relation with the plastic changes for the Hcrt/OxR2, a great variability took place after administration of the low dose in the two Hcrt/OxR2 bands (see Figures 3A,B and Supplementary Figure 2), but both bands showed a similar trend with increased levels for the low dose but not for the high one (Figures 3A,B). The corresponding Krustal–Wallis analyses of variance indicated that the changes were more pronounced for the upper band, with almost significant differences (*H* = 5.361, *p* = 0.068), than for the lower band (*H* = 4.309, *p* = 0.116). *Post hoc* comparisons showed that the main differences for the Hcrt/OxR2 upper band occurred between doses (*U* test: *Z* = −2.119, *p* = 0.034) (Figure 3A). Comparisons between control and the low dose groups showed only nearly significant differences (*U* test: *Z* = 1.841, *p* = 0.066) while no significant values appeared between the high dose of suvorexant and the control groups (*U* test: *Z* = 0.475, *p* = 0.635).

Finally, regarding the body weight changes and the molecular variations in the Hcrt/Ox system analyzed here in relation with the sex of the animals, Table 1 summarizes the results of the two-way ANOVAs, for the normalized percentages of those changes between the control and the high-dose of suvorexant groups (treatment factor), considering the values of males and females (sex factor). Note from this table the statistically significant changes produced by the treatment with the high dose of suvorexant in all the physiological and molecular variables except for hypothalamic Hcrt/OxR2 values. In contrast, there were no statistically significant changes in relation with sex factor. However, the interaction “treatment × sex” resulted to reach statistically significant values for the hypothalamic Hcrt/OxR1 levels.

**TABLE 1:** Sex-related effects of suvorexant treatment in physiological and molecular variables.

Variable	Treatment factor		Sex factor		Interaction treatment × Sex	
Body weight (days 7–10)	*F*_(1,15)_ = 8.200	*p* ≤ 0.025	*F*_(1,15)_ = 4.648	*p* ≤ 0.10	*F*_(1,15)_ = 0.060	*p* > 0.25
CSF Hcrt1/OxA levels	*F*_(1,15)_ = 7.721	*p* ≤ 0.025	*F*_(1,15)_ = 0.232	*p* > 0.25	*F*_(1,15)_ = 0.960	*p* > 0.25
Hypothalamic Hcrt/OxR1 levels	*F*_(1,13)_ = 7.760	*p* ≤ 0.025	*F*_(1,13)_ = 0.316	*p* > 0.25	*F*_(1,13)_ = 6.356	*p* ≤ 0.025
Hypothalamic levels of Hcrt/OxR2 upper band	*F*_(1,15)_ = 0.334	*p* > 0.25	*F*_(1,15)_ = 0.531	*p* > 0.25	*F*_(1,15)_ = 2.293	*p* ≤ 0.25
Hypothalamic levels of Hcrt/OxR2 lower band	*F*_(1,15)_ = 2.040	*p* ≤ 0.25	*F*_(1,15)_ = 0.028	*p* > 0.25	*F*_(1,15)_ = 0.064	*p* > 0.25

Two-way ANOVAs comparing changes between the control and the high-dose of suvorexant groups for the different physiological and molecular variables.

## Discussion

Dual Hcrt/Ox receptor antagonists produce increases in both slow-wave and REM sleep (Winrow et al., 2011; Gotter et al., 2014; Mahoney et al., 2020; Kaushik et al., 2021). Furthermore, a 7-day chronic blockade of Hcrt/Ox transmission with dual orexin receptor antagonists (DORAs) in wild type mice (Mahoney et al., 2020; Kaushik et al., 2021) produces REM sleep-related narcoleptic symptoms -such as cataplexy- after exposure to certain stimuli, as is observed in human NT1 (Schiappa et al., 2018). In our experiments in rats we administered two doses –10 and 30 mg/kg- of the drug daily, since it is known that in the rat a 30 mg/kg dose is sufficient to produce changes related to sleep and locomotor activity comparable with those induced with higher doses (Winrow et al., 2011).

To evaluate the effectiveness of the chronic treatment with suvorexant in our rats, we analyzed the changes in the body weight of the animals throughout the treatments. Regardless of the experimental group, a moderate but statistically significant decrease in body weight at the end of the treatments occurred. Nevertheless, only the control group presented and maintained a decrease throughout the whole experimental period, whereas the animals receiving suvorexant showed a recovery in their body weight at the end of the treatment. Furthermore, for the high-dose group the increase proved to be statistically significant when compared with the control group. In agreement with the increase in body-mass index observed in narcoleptic patients (Schuld et al., 2000; Dahmen et al., 2001; Kok et al., 2003), our results point to the effectiveness of suvorexant high-dose in mimicking some phenotypic traits of narcolepsy.

Concerning the molecular changes in the Hcrt/Ox system induced by suvorexant, another common feature between the results of the present experiments in rats and human NT1 is the significant decrease in CSF Hcrt1/OxA levels observed in the high-dose group by comparison with the control and the low-dose groups. The decrease in CSF Hcrt1/OxA levels constitutes a more specific biomarker of NT1 than any other such as hypersomnolence, or even cataplexy (American Academy of Sleep Medicine [AASM], 2014). Our results agree with those of Kaushik et al. (2021) reporting a decrease of Hcrt1/OxA levels in the whole brain tissue of mice after a similar suvorexant treatment. Although we have not evaluated cataplexy-like behavior and sleep pattern in our rats as it has been done in mice (Mahoney et al., 2020; Kaushik et al., 2021), it seems that the 30 mg/kg dose of suvorexant in the rat experimental model is able to reproduce a functional situation present in NT1 patients, despite the fact that the cells of the Hcrt/Ox system would be undamaged. We believe that from the cellular and molecular point of view, the decrease in CSF Hcrt1/OxA concentration observed in our pharmacological model after the systemic blockade of both Hcrt/Ox receptors strongly supports the existence of a positive feedback loop necessary for Hcrt/Ox peptide expression and/or release.

It is well known that in the posterior hypothalamus, besides the Hcrt/Ox cells, there is also a dense innervation of Hcrt/Ox axons (Peyron et al., 1998; Horvath et al., 1999; Van Den Pol et al., 2001). In fact, the Hcrt/Ox cells themselves are innervated by Hcrt/Ox fibers (Horvath et al., 1999; Yamanaka et al., 2010), therefore it has been proposed that the Hcrt/Ox cells can exert their own self-regulatory actions regardless of those that may be mediated by other intra- and extra-hypothalamic neurotransmission systems also afferent to Hcrt/Ox cells (Burt et al., 2011). Also, it has been suggested that this self-regulation could be performed by Hcrt/OxR1, whose presence in the perikarya of Hcrt/Ox cells was immunocytochemically demonstrated (Bäckberg et al., 2002). Furthermore, since canine familial narcolepsy is caused by a mutation in the gene encoding Hcrt/OxR2 (Lin et al., 1999), and narcoleptic dogs exhibited unaltered levels of Hcrt1/OxA in CSF (Ripley et al., 2001), the Hcrt/OxR2 seems not to play an important role in the synthesis and/or release of Hcrt/Ox. However, it has been suggested that the Hcrt/OxR2 could be involved in the depolarization of Hcrt/Ox neurons by Hcrt/Ox fibers in *in vitro* experiments (Yamanaka et al., 2010) although the presence of the Hcrt/OxR2 has not been reported in the Hcrt/Ox cells (Vassalli et al., 2015).

In our study, the control group of rats presented hypothalamic Hcrt/OxR1 levels significantly higher than those of Hcrt/OxR2, suggesting an important role for the former within this area. Kaushik et al. (2021) reported that in the whole brain tissue of mice, Hcrt/OxR1 mRNA levels did not change after chronic suvorexant treatment, but there was an initial decrease of Hcrt/OxR2 mRNA levels followed by an increase at the end of the treatment. To our knowledge, no plastic changes regarding the Hcrt/Ox receptors have been specifically evaluated in the hypothalamus so far. When analyzing the expression of the Hcrt/Ox receptors at the protein level, our study shows that specifically in the hypothalamus, the chronic treatment with the high dose of suvorexant produced a significant overexpression of Hcrt/OxR1 in parallel with the decrease in CSF Hcrt1/OxA, but no expression changes were observed regarding the Hcrt/OxR2. In concert with above reported Hcrt/Ox fibers in contact with Hcrt/Ox neurons (Horvath et al., 1999; Yamanaka et al., 2010), the expression of Hcrt/OxR1 within the Hcrt/Ox perikarya (Bäckberg et al., 2002) and the normal concentration of Hcrt1/OxA in CSF in narcoleptic dogs, our results provide a conclusive support for the role of Hcrt/OxR1 as an autoreceptor in the Hcrt/Ox system. Furthermore, the Hcrt/OxR1 sequence is highly conserved among mammals but absent in the genomes of species outside that group (Soya and Sakurai, 2020), and the Hcrt/OxR1 is selective for Hcrt1/OxA binding. On the contrary, the Hcrt/OxR2 sequence shows some variations and binds indistinctly Hcrt1/OxA and Hcrt2/OxB (Sakurai et al., 1998; Wang et al., 2018). This also indicates a strong evolutionary pressure for maintaining the signaling mediated by the binding of Hcrt1/OxA to Hcrt/OxR1 in order to preserve the proper Hcrt/Ox system operation in the phylogenetic group of mammals.

**FIGURE 5:**
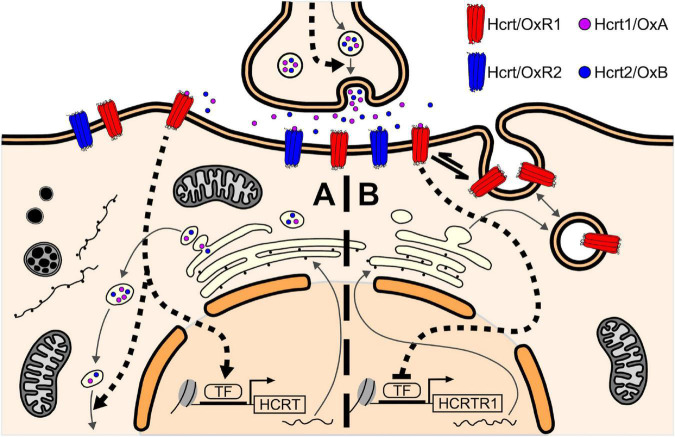
Self-regulatory model of the Hcrt/Ox system mediated by Hcrt/OxR1 within the hypothalamic Hcrt/Ox neurons. **(A)** Hcrt1/OxA binding to Hcrt/OxR1 in the Hcrt/Ox neurons would activate downstream signaling pathways that induce the expression of the HCRT gene and/or Hcrt/Ox peptides release (dashed arrows), thus establishing a positive feedback loop for Hcrt/Ox peptide synthesis/release. **(B)** On the other hand, Hcrt1/OxA binding to Hcrt/OxR1 would also activate signaling cascades that block the expression of the HCRTR1 gene (blunted-head dashed arrow) and/or induce the internalization of available Hcrt/OxR1 on the plasma membrane to intracellular vesicles (equilibrium arrow), thus establishing a negative feedback loop for the availability of Hcrt/OxR1. This self-regulating function of the receptor, which could also be present in other neuronal groups, would counteract the positive feedback loop for Hcrt/Ox peptide synthesis, generating a homeostatic balance between them. TF, transcription factor.

All of the above enables us to propose a model of self-regulation of the Hcrt/Ox system mediated by Hcrt/OxR1 within the hypothalamus (Figure 5). On the one hand the Hcrt/OxR1 would induce the expression of the gene encoding Hcrt/Ox peptide and/or Hcrt/Ox peptides release (Figure 5A), and on the other hand it would block its own expression (Figure 5B). According to this model, the blockade of the Hcrt/Ox signaling, and more specifically that mediated by Hcrt/OxR1, would produce a decrease in the CSF Hcrt1/OxA levels together with a compensatory increase in the expression of Hcrt/OxR1. This prediction agrees with the observations in our animals treated with the high dose of suvorexant.

With regard to Hcrt/OxR2, the two different but very close bands observed in the western blot membrane could correspond to the two previously described isoforms of the Hcrt/OxR2 in the mouse (Chen and Randeva, 2004), both expressed in the hypothalamic tissue (Chen et al., 2006), that may be also present in the rat. The upper band would correspond to the largest splice variant (460aa), while the lower band would correspond to the smallest one (443aa). No significant changes were found for the two bands when comparing the group treated with the high dose of suvorexant and the control group. For the low-dose group an increase in the levels of both Hcrt/OxR2 bands was observed, which in fact was statistically significant for the upper band when compared to the high-dose group. Distinct effects for high and low antagonist doses have also been described in other neurotransmission systems with the mediation of GABAergic neurons (Dos Santos Dantas et al., 2006; Hiyoshi et al., 2014). Since the GABAergic neurons of various hypothalamic nuclei have been reported to be activated by hypocretins (Burdakov et al., 2003; Eriksson et al., 2004), more complex signaling pathways, probably involving GABAergic neurons, must participate in the regulation of hypothalamic Hcrt/OxR2 expression.

In relation with male/female effects produced by the treatment with suvorexant, the trend was the same for both sexes. However, the interaction of treatment and sex factors resulted significant for the levels of Hcrt/OxR1, thus indicating that female rats had a potentiation in the response. Nevertheless, no sex-related significant differences occurred for the decrease of Hcrt1/OxA levels in CSF or for the plastic changes regarding Hcrt/OxR2. The increment of body weight by the end of the treatment -although near- did not show sex significant values either. Previous studies have indicated higher expression levels of both Hcrt/Ox peptides and their receptors in female compared to male rats (Taheri et al., 1999; Jöhren et al., 2001, 2002), which vary throughout the estrous cycle (Silveyra et al., 2007). Furthermore, a higher activation of Hcrt/Ox neurons in females has been described (Grafe et al., 2017). This increased Hcrt/Ox activity in female rats, which positively correlates with estrogen plasma levels (Grafe and Bhanagar, 2020), would explain a greater severity of its blockade compared to males in our experiments. Although, in fact, the mechanisms underlying sex differences with respect to the Hcrt/Ox system remain poorly understood. The study of sex differences in relation with the Hcrt/Ox system deserves especial attention not only for their molecular and cellular characteristics, but also for the knowledge of their physiological and behavioral effects.

In conclusion, this study shows that the chronic systemic blockade of Hcrt/Ox receptors with a 30 mg/kg dose of suvorexant in rats generates a pharmacological model that shares some important features of human NT1. These include a significant increase in body weight at the end of the treatment, and a significant decrease in CSF Hcrt1/OxA levels likely due to the blockade of Hcrt/OxR1. Our results give a conclusive support to the role of the Hcrt/OxR1 as an autoreceptor suggested previously by other authors. Our findings are key to get insight into adaptation mechanisms of Hcrt/Ox neurons during progressive degeneration in narcolepsy. The Hcrt/OxR1 would regulate Hcrt/Ox peptide circulating levels, as well as its own receptor expression, as a homeostatic self-regulatory mechanism of the Hcrt/Ox system. None of the two possible isoforms of Hcrt/OxR2 seems to have an important function in hypothalamic self-regulatory Hcrt/Ox mechanisms. However, the Hcrt/OxR2 could be responsible for important actions in the Hcrt/Ox system at the level of their target cells in central nervous regions related to the mechanisms of the sleep-wake cycle (Lin et al., 1999; Ripley et al., 2001; Willie et al., 2003; Irukayama-Tomobe et al., 2017; Yukitake et al., 2019). Nonetheless, the Hcrt/OxR1 in its role as autoreceptor inducing the synthesis and/or release of the Hcrt/Ox peptides would have a pivotal role not only in sleep mechanisms, but also in all the physiological functions mediated by the Hcrt/Ox system.

Finally, we believe that the experiments with chronic systemic administration of suvorexant in rats lead to a pharmacological model, in which the Hcrt/Ox cells would remain undamaged, that mimics several traits of narcolepsy. We think that this would be a good experimental model for the study of the cellular and molecular neurobiological bases of the wide range of Hcrt/Ox system functions in wild-type animals; and especially for the experimental study of narcoleptic traits and REM sleep mechanisms using cats and rats, the species where knowledge of sleep mechanisms have been accumulated for years (Reinoso-Suárez et al., 2001; Moreno-Balandrán et al., 2008; Sánchez-López et al., 2018; Carrera-Cañas et al., 2019).

## Data availability statement

The raw data supporting the conclusions of this article will be made available by the authors, without undue reservation.

## Ethics statement

The animal study was reviewed and approved by Comité de Ética de la Investigación, Universidad Autónoma de Madrid.

## Author contributions

CC-C, IA, and MG: conceptualization, and writing – original draft preparation and review and editing. CC-C and MC: methodology. CC-C, IA, MC, and MG: validation and investigation. CC-C and IA: formal analysis. All authors have read and agreed to the published version of the manuscript.
