# Supplementary material for: Plasticity of the hypocretinergic/orexinergic system after a chronic treatment with suvorexant in rats. Role of the hypocretinergic/orexinergic receptor 1 as an autoreceptor
Source: Front Mol Neurosci. 2022 Oct 5;15:1013182. doi: 10.3389/fnmol.2022.1013182 (PMC9581150; doi:10.3389/fnmol.2022.1013182)
Supplement: Supplementary file 1 [file Presentation_1.PPTX]

## Slide 1
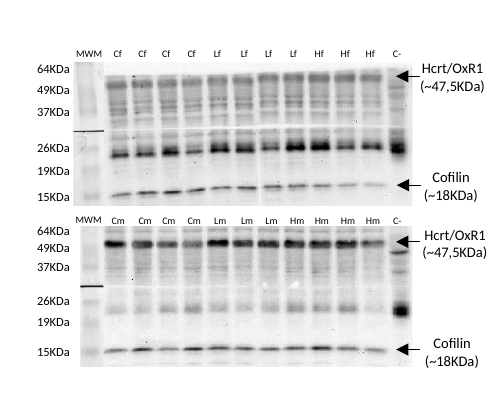

MWM
Lf
Lf
Lf
Lf
Hf
Hf
Hf
C-
Cf
Cf
Cf
Cf
Hcrt/OxR1 (~47,5KDa)
64KDa
49KDa
37KDa
26KDa
19KDa
Cofilin (~18KDa)
15KDa
MWM
Lm
Lm
Lm
Hm
Hm
Hm
Hm
C-
Cm
Cm
Cm
Cm
64KDa
Hcrt/OxR1 (~47,5KDa)
49KDa
37KDa
26KDa
19KDa
Cofilin (~18KDa)
15KDa
