# Supplementary material for: Plasticity of the hypocretinergic/orexinergic system after a chronic treatment with suvorexant in rats. Role of the hypocretinergic/orexinergic receptor 1 as an autoreceptor
Source: Front Mol Neurosci. 2022 Oct 5;15:1013182. doi: 10.3389/fnmol.2022.1013182 (PMC9581150; doi:10.3389/fnmol.2022.1013182)
Supplement: Supplementary file 2 [file Presentation_2.PPTX]

## Slide 1
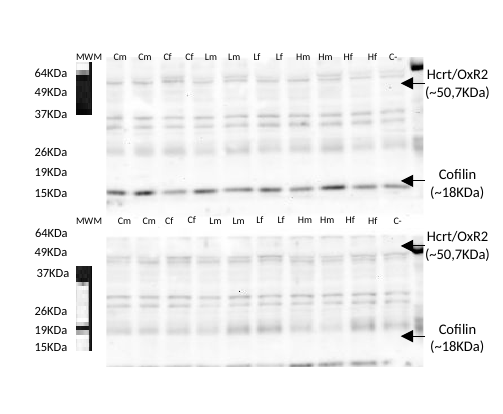

MWM
Lm
Lm
Lf
Lf
Hm
Hm
Hf
Hf
C-
Cm
Cm
Cf
Cf
Lf
Lf
Hm
Hm
Hf
Cf
Lm
Hf
C-
Lm
Cm
Cm
Cf
MWM
64KDa
49KDa
37KDa
26KDa
19KDa
15KDa
64KDa
49KDa
37KDa
26KDa
19KDa
15KDa
Hcrt/OxR2 (~50,7KDa)
Cofilin (~18KDa)
Hcrt/OxR2 (~50,7KDa)
Cofilin (~18KDa)
